# Apoptosis related genes mediated molecular subtypes depict the hallmarks of the tumor microenvironment and guide immunotherapy in bladder cancer

**DOI:** 10.1186/s12920-023-01525-8

**Published:** 2023-04-28

**Authors:** Liquan Zhou, Guanglong Xu, Fu Huang, Wenyuan Chen, Jiange Zhang, Yong Tang

**Affiliations:** 1 Department of Urology, The Second Affiliated Hospital, Guangxi Medical University, Nanning, 530006 Guangxi China; 2 Department of Urology, Wuming Hospital, Guangxi Medical University, Nanning, 530199 Guangxi China

**Keywords:** Bladder cancer, Apoptosis, Tumor microenvironment, Immunotherapy, Scoring system

## Abstract

Apoptosis has been discovered as a mechanism of cell death. The purpose of this study is to identify the diagnostic signature factors related to bladder cancer (BLCA) through apoptosis related genes (ARGs). Clinicopathological parameters and transcriptomics data of 1,440 BLCA patients were obtained from 7 datasets (GSE13507, GSE31684, GSE32548, GSE32894, GSE48075, TCGA-BLCA, and IMvigor210). We first identified prognosis-related ARGs in BLCA and used them to construct two ARGs molecular subtypes by using consensus clustering algorithm. By using principal component analysis algorithms, a ARGscore was constructed to quantify the index of individualized patient. High ARGscore correlated with progressive malignancy and poor outcomes in BLCA patients. High ARGscore was associated with higher immune cell, higher estimate scores, higher stromal scores, higher immune scores, higher immune checkpoint, and lower tumor purity, which was consistent with the “immunity tidal model theory”. Preclinically, BLCA immunotherapy cohorts confirmed patients with low ARGscore demonstrated significant therapeutic advantages and clinical benefits. These findings contribute to our understanding of ARGs and immunotherapy in BLCA. The ARGscore is a potentially useful tool to predict the prognosis and immunotherapy in BLCA.

**Supplementary Information:**

The online version contains supplementary material available at 10.1186/s12920-023-01525-8.

## Introduction

Bladder cancer (BLCA) is a major contributor to cancer-related deaths [1]. BLCA can present as non-muscle-invasive BLCA, muscle-invasive BLCA, and metastatic disease. Currently, the efficacy of BLCA treatment strategies is not satisfactory due to high heterogeneity and drug resistance [2, 3]. Immune checkpoint inhibitors (ICIs) have shown promising therapeutic results in BLCA [4, 5]. However, due to differences in individual immunogenicity and tumor microenvironments (TME), the response rate to this therapy in patients without selective treatment is not satisfactory. Meanwhile, the biomarkers of ICIs has certain limitations [6, 7]. Thus, it is important to develop immunotherapy biomarkers from a genomic perspective to meet the needs of individualized therapy for patients with different genetic backgrounds.

Recent studies have shown that apoptosis regulates tumorigenesis and progression extensively [8]. Tumor development and immune response are all influenced by the apoptosis signaling pathway [9]. In response to stimulation by appropriate external or internal signals, cells may alter the expression of genes encoding proteins associated with the apoptotic process. Alteration of genes associated with apoptosis in bladder cancer leads to a shift from a default apoptotic state to drug-resistant cells with higher survival characteristics [10]. The TME contains tumor cells, vasculature, extracellular matrix (ECM), stromal, and immune cells [11–13]. In vitro and in vivo experiments have also shown that apoptotic tumor cells induce antitumor immunogenicity by cross-initiating and proliferating CD8 T cells [14, 15].

Therefore, this study focuses on the functional role of apoptosis related genes (ARGs) in BLCA. The purpose of this study is to explore the expression of ARGs in BLCA by bulk RNA sequencing, and to identify their expression. To further investigate the effect of ARGs expression in BLCA on the development of BLCA, and to explore its influence on the biological behavior of BLCA, and to provide a basis for the etiology, pathogenesis and prevention of primary BLCA. Meanwhile, we adopted the consensus clustering algorithm to construct the consensus matrix and cluster the samples’ BLCA related expression profile data. Then, a ARGscore was constructed to quantify the apoptosis index of individualized patient. Our study might provide clues for targeted therapeutics in BLCA.

## Materials and methods

### Data collection and pre-processing

Clinicopathological parameters and transcriptomics data were obtained from nine independent BLCA cohorts (GSE13507 [16], GSE31684 [17], GSE32548 [18], GSE32894 [19], GSE48075 [20], TCGA-BLCA, and IMvigor210 [21]). The FPKM of BLCA and normal tissues were downloaded from TCGA database website, and the raw data were extracted into matrix files by Perl software. The correspondence between human gene names and gene ids was downloaded from ensemble website, and the gene ids in the raw data were converted into gene names by Perl software. Then, the FPKM values are converted to TPM values. The TCGA data and GEO data are integrated into a meta cohort using the "sva" package. The data information is summarized in Table 1 and Additional file 1: Table S1.

**Table 1:** Basic information of BLCA cohorts included in this study

Source	Platform	Platforms	Number of patients	Prognosis data	Notes
GSE13507	Illumina human-6 v2.0 expression beadchip	GPL6102	165	Yes	Training; Internal validation
GSE31684	Affymetrix Human Genome U133 Plus 2.0 Array	GPL570	93	Yes	Training; Internal validation
GSE32548	Illumina HumanHT-12 V3.0 expression beadchip	GPL6947	130	Yes	Training; Internal validation
GSE32894	Illumina HumanHT-12 V3.0 expression beadchip	GPL6947	224	Yes	Training; Internal validation
GSE48075	Illumina HumanHT-12 V3.0 expression beadchip	GPL6947	73	Yes	Training; Internal validation
TCGA-BLCA	–	–	407	Yes	Training; Internal validation
IMvigor210	–	–	348	Yes	External validation

*TCGA* The Cancer Genome Atlas, *BLCA* Bladder cancer

### Expression and prognosis of ARGs in BLCA

We obtain 91 ARGs from the previous studies (Additional file 2: Table S2) [22]. Then, we compared ARGs expression in TCGA-BLCA (|log FC|≥ 1.0 and adj. P < 0.05) by using “limma” package [23]. Univariate Cox regression analyses were performed by “survival” packages to acquire prognostic-related ARGs [24].

### Consensus clustering analysis

In order to better classify the ARGs into different clusters, we adopted the ConsensusClusterPlus tool to construct the consensus matrix and cluster the ARG related expression profile data of samples. We adopted the "KM" algorithm and "1-Pearson correlation", and applied the metric distance and 500 bootstraps, with each bootstrap process including 80% of patients in the training set. We identified the cluster number as 2 to 10. The number of clusters (K) is chosen by the maximum average profile value [25].

### Identification of DEGs and prognosis genes between ARGs subtypes

Different expression of ARGs were analyzed in 2 ARGs subtypes using the "limma" package, and the screening criteria were | log2Fold Change (FC)|> 1, FDR < 0.05. Univariate Cox regression analyses were performed by “survival” packages to acquire prognostic-related DEGs.

### GO and KEGG enrichment analysis

To explore the biological functions mainly performed by DEGs and prognosis genes between ARGs subtypes, then the up-regulated and down-regulated genes were analyzed for GO and KEGG enrichment in metascape [26–28]. The screening criteria were P < 0.05 and FDR < 0.05[29].

### Evaluation of immune infiltrating cells in BLCA

The ESTIMATE algorithm can calculate the immune and stromal fractions of the tumor microenvironment of tumor samples, and a larger fraction indicates a larger proportion of immune cells or stromal cells in the tumor microenvironment. ssGSEA and TIMER were utilized to reveal the immune cell infiltration patterns, determine the proportions of circulating immune cells and analyze the correlation between these immune cells [30].

### Tracking tumor immunophenotype analysis

Tracking Tumor Immunophenotype (TIP) analysis was done according to website protocol. Transcriptomic expression profile was used as the input for analysis [31].

### Construction of ARGscore

Based on the DEGs and prognosis genes between ARGs subtypes, we established a ARGscore by using PCA method [32–35]. The final ARGscore was calculated as follows:

$$\mathrm{ARGscore }=\Sigma \left(P\mathrm{C}{1}_{i}+P\mathrm{C}{2}_{i}\right),$$

where i is the expression value of each gene in the signature. A high ARGscore group and a low ARGscore group were divided base on the median ARGscore.

### GSEA analysis, clinical relevance, and immune correlation analysis of ARGscore

The differentially expressed genes in the ARGscore high and low groups in liver cancer samples were analyzed by GSEA to explore the impact of the difference in ARGscore levels on cell signaling pathways. Enrichment analysis was performed according to the default reference settings, and q < 0.05 was selected as the set of significantly enriched genes [36].

### Immunohistochemistry (IHC) staining

IHC was performed on formalin fixed, paraffin-embedded tissue sections using a two-step protocol. Then using traditional method to evaluate under the optical microscope (N = 10). The paired BLCA tissues and non-tumor tissues were collected from the patients at Wuming Hospital, Guangxi Medical University. This study was evaluated and reviewed by the Ethics Committee of Wuming Hospital, Guangxi Medical University (WM-2022-15). Written consent was obtained from all patients prior to the study.

## Statistical methods

Data analysis and graphing were carried out using R 4.2.2 software. The Wilcoxon assay was used to assess the difference in ARGs between normal and tumor tissues. The association between ARGs regulators expression and prognosis was evaluated using univariate Cox regression analysis. Wilcoxon analysis was performed to assess the correlation between ARGscore and immune cell infiltration levels and immune checkpoint inhibitors expression. The predictive efficiency of the ARGscore model was tested by the ROC curve. The difference was considered statistically significant at P < 0. 05 (*P < 0.05; **P < 0.01; ***P < 0.001; ****P < 0.0001).

## Results

### Differential expression and prognosis of ARGs

**Fig. 1:**
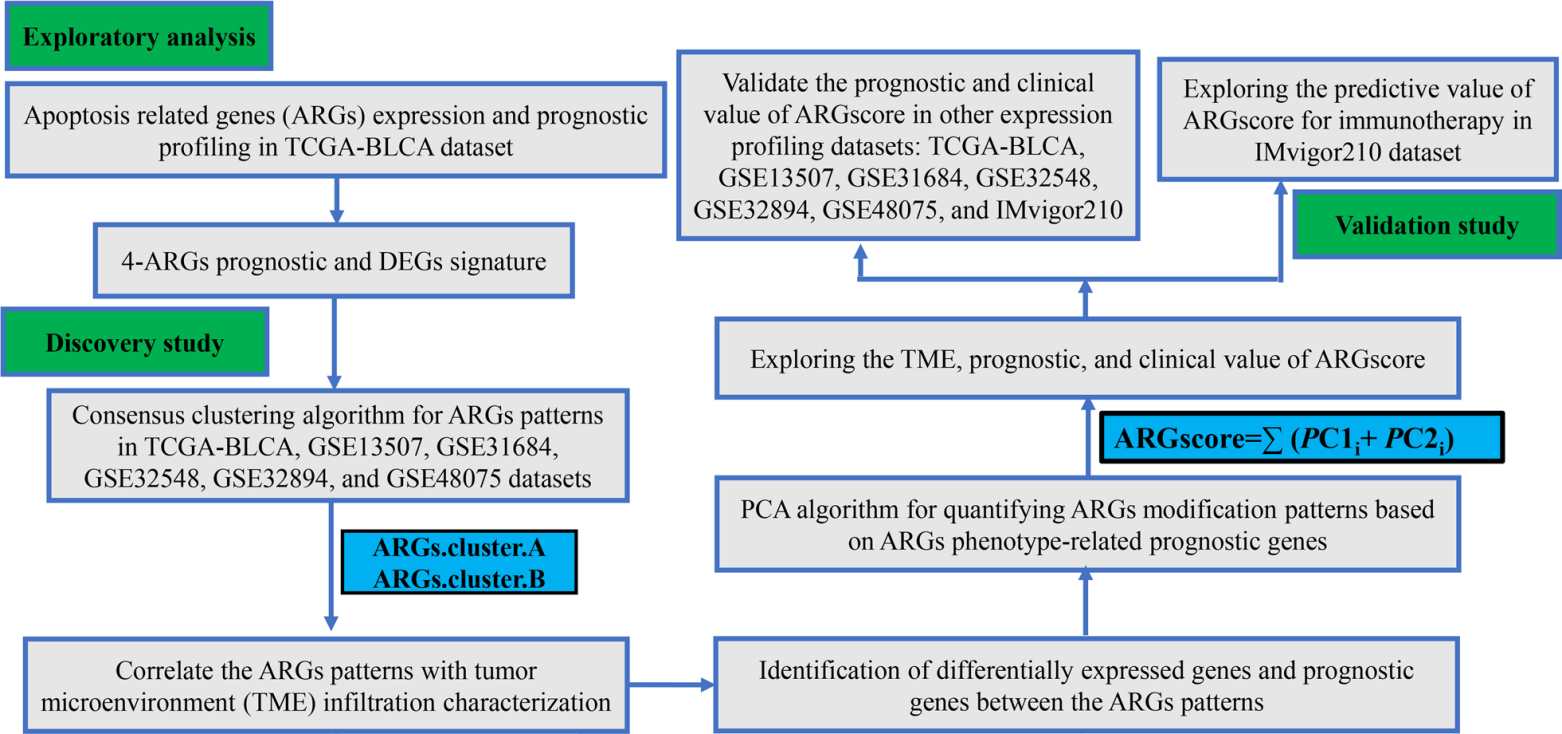
Flow chart of the study

**Fig. 2:**
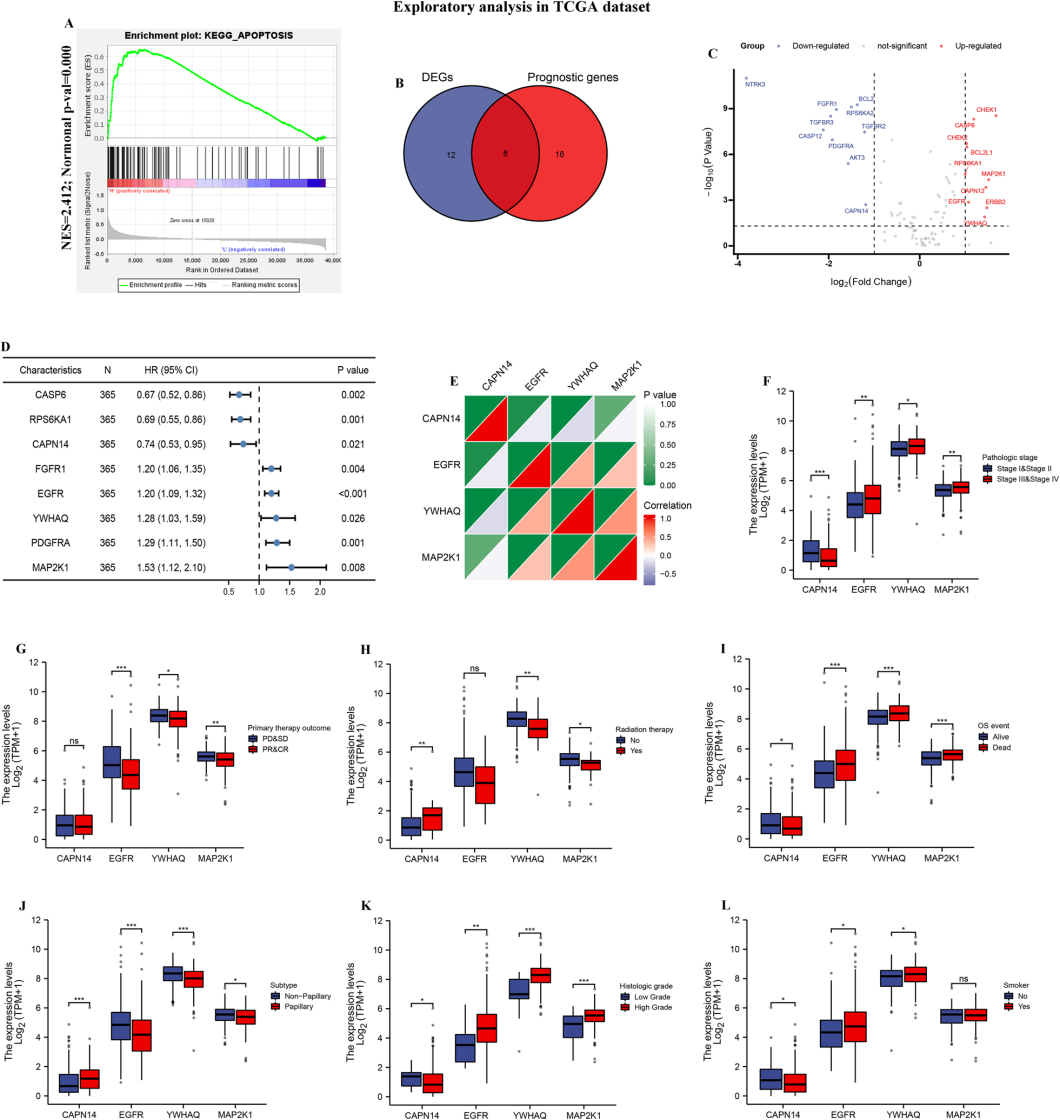
Identification of differential expression and prognostic of ARGs in TCGA-BLCA. **A** GSEA showed that the apoptosis pathway are differentially enriched in BLCA patients. **B** Venn diagram to identify differentially expressed genes between tumor and normal tissue that were correlated with overall survival. **C** Volcano plot of differentially expressed ARGs between tumor and normal tissue. Dot stands for gene. Red dots represent up-regulated genes. **D** Forest plots showing the results of the univariate Cox regression between 8 ARGs and overall survival in BLCA. **E** Correlation heat map of 4 ARGs. **F** The illustration shows the expression distribution of 4 ARGs between stage 3&4 (red) and stage 1&2 (blue). **G** The illustration shows the expression distribution of 4 ARGs between PR&CR group (red) and PD&SD group (blue). **H** The illustration shows the expression distribution of 4 ARGs between radiation therapy group (red) and no radiation therapy group (blue). **I** The illustration shows the expression distribution of 4 ARGs between dead (red) and alive (blue). **J** The illustration shows the expression distribution of 4 ARGs between papillary group (red) and non-papillary group (blue). **K** The illustration shows the expression distribution of 4 ARGs between high grade group (red) and low grade (blue). **L** The illustration shows the expression distribution of 4 ARGs between smoker group (red) and no smoker group (blue). (*P < 0.05; **P < 0.01; ***P < 0.001)

The workflow of our analysis was shown in Fig. 1. Firstly, GSEA showed that apoptosis pathway was significant enriched in the BLCA (Fig. 2A). Then, we compared the expression of 91 ARGs, of which the expression level of 10 ARGs were significantly higher in BLCA than in the normal tissues (Fig. 2B, C, Additional file 3: Table S3). In total, 219 nonsmokers and 59 smokers had adequate knowledge of age, sex, grade, clinical staging, survival status, and follow-up time. The mean age of all participants at diagnosis was 68.97 ± 10.57 years, with 71.22% of male participants, almost 2.5 times more than females. Next, univariate Cox regression analysis was performed based on TCGA data, 24 ARGs coorelated with outcome in BLCA (Fig. 2D, Additional file 4: Table S4). Based on these results, 4 ARGs (*CAPN14, EGFR, YWHAQ, MAP2K1*) were considered as risk genes. The 4 ARGs had positive correlation with each other (Fig. 2E). Moreover, *CAPN14, EGFR, YWHAQ, MAP2K1* expression were higher in stage III & stage IV than stage I & stage II, higher in radiation therapy group than in no radiation therapy group, higher in PD&SD group than in PR&CR, higher in dead group than in alive, higher in non-papillary group than in papillary, higher in high Grade group than in low Grade, higher in smoker group than in no smoker (Fig. 2F–L). Meanwhile, these four genes were significantly associated with immune infiltration (Additional file 8: Fig. S1A–D). The above results suggest that these 4 ARGs may be associated with the metastasis of BLCA.

### Identification of ARGs subtypes in BLCA

**Fig. 3:**
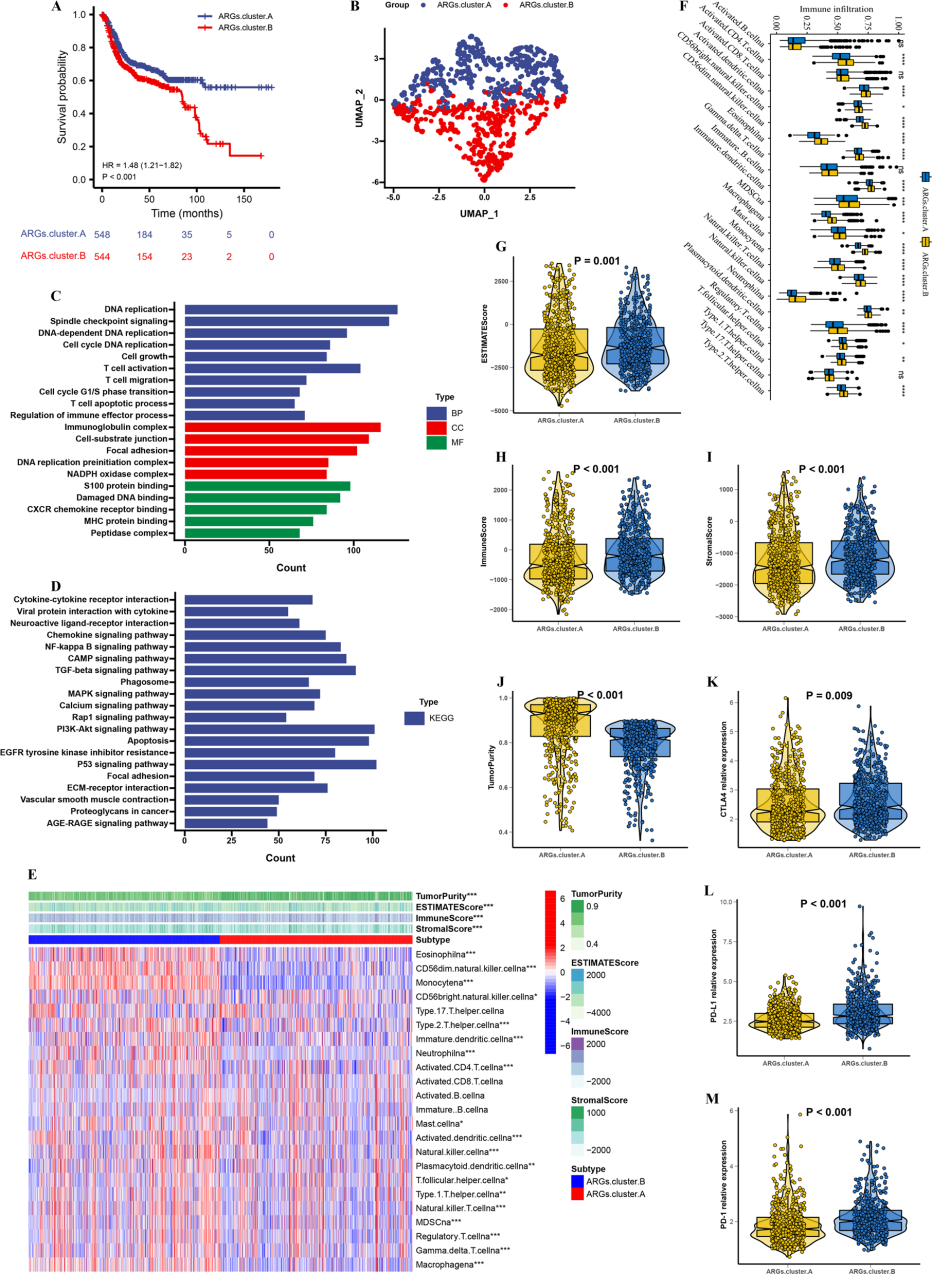
The molecular subtypes categorization of BLCA base on 4 ARGs. **A** Kaplan–Meier curve showed a significant difference between the two ARGs clusters. **B** UMAP analysis for the transcriptome profiles of ARGs.cluster.A and ARGs.cluster.B, showing a remarkable difference on transcriptome between different group. **C** GO enrichment analysis, **D** KEGG enrichment analysis for the different expression that were correlated with OS genes. **E** The correlation of tumor microenvironment condition and ARGs.cluster. **F** The abundance of each TME infiltrating cell in two ARGs.clusters. The upper and lower ends of the boxes represented the interquartile range of values. The lines in the boxes represented median value, and black dots showed outliers. **G**–**J** The ESTIMATE score, stromal score, immune score, and tumor immunity levels in the ARGs.cluster.A and ARGs.cluster.B groups by using ESTIMATE algorithm. **K**–**M** The PD-1, PD-L1 and CATA4 levels in the ARGs.cluster.A and ARGs.cluster.B groups. (*P < 0.05; **P < 0.01; ***P < 0.001; ****P < 0.0001)

Then, we adopted the ConsensusClusterPlus tool to construct the consensus matrix and cluster the 4 ARGs related expression profile data of samples. The unsupervised method NMF analysis results in 2 subtypes ARGs.cluster.A (n = 548) and ARGs.cluster.B (n = 544) (Additional file 8: Fig. S2A–F). The overall survival (OS) time in ARGs.cluster.A group was significantly longer than that in ARGs.cluster.B group (HR = 1.48, P < 0.001, Fig. 3A). The PCA demonstrated there is significant DEGs between the two clusters, and 2859 prognostic ARGs-related DEGs were identified (Fig. 3B, Additional file 5: Table S5). Then, we applied GO and KEGG, which showed that ARGs.cluster.B was significantly enriched in immune-related pathways (T cell activation, T cell migration, T cell apoptotic process, MHC protein binding, and cytokine-cytokine receptor interaction) and cancer-related pathways (DNA replication, cell growth, apoptosis, and PI3K-Akt signaling pathway) (Fig. 3C, D), suggesting that ARGs.cluster.B may play an important role in tumor development and immune regulation.

In order to clarify the differences in TME between the 2 established clusters, we investigated the gene expression and infiltration level of immune cells by ssGSEA. The results suggested that the infiltration level of most immune cells was significantly lower in ARGs.cluster.A group (Fig. 3E,F). The ESTIMATE results showed that the stromal score, immune score and ESTIMATE score in ARGs.cluster.B group were much higher than those in ARGs.cluster.A group (Fig. 3G–J). Immune checkpoint blockade-mediated immunotherapy has shown promising effects against tumors, and has proved as effective in a significant proportion of refractory patients undergoing standard therapy. In this analysis, we evaluated some representative targets, and found that immune checkpoint were highly expressed in ARGs.cluster.B group (Fig. 3K–M, Additional file 8: Fig. S3). The above results suggest that ARGs.cluster.B was consistent with the “immunity tidal model theory”, thus leading to a worse prognosis for ARGs.cluster.B.

### Construction of ARGscore

**Fig. 4:**
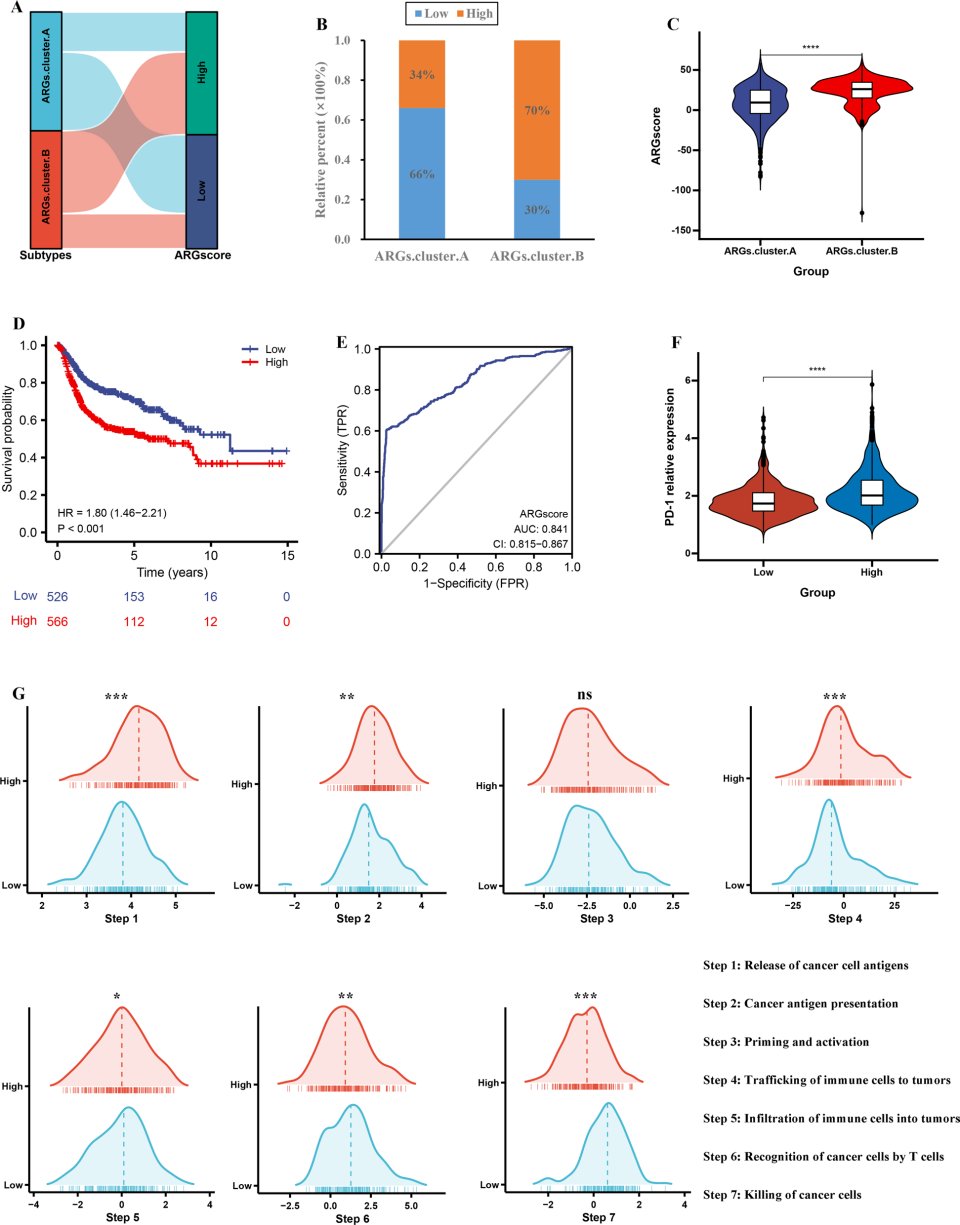
Construction of ARGscore. **A** Alluvial diagram showing the changes of ARGs.cluster and ARGscore. **B** The proportion of patients with high and low ARGscore in ARGs.cluster.A and ARGs.cluster.B groups. **C** Differences in ARGscore among two ARGs.cluster. **D** Kaplan–Meier curves for high and low ARGscore groups. **E** The predictive value of ARGscore. **F** The PD-1 levels in the high and low ARGscore groups. **G** Differences in various steps of Cancer-Immunity Cycle. (*P < 0.05; **P < 0.01; ***P < 0.001; ****P < 0.0001)

A ARGscore was constructed based on 2859 prognostic ARGs-related DEGs. Alluvial diagram confirmed the relationship between the ARGs.cluster and ARGscore (Fig. 4A). ARGs.cluster.B group had a higher proportion of high ARGscore (Fig. 4B). Next, we analyzed the distribution of ARGscore in the ARGs.cluster, and ARGscore was lower in the ARGs.cluster.A group (Fig. 4C). The results also showed that patients with high ARGscore had a worse overall survival (HR = 1.80, P < 0.001, Fig. 4D). To test the prediction performance of ARGscore, we plotted ROC curves with AUC values of 0.841, suggesting ARGscore had a good predictive performance (Fig. 4E). In addition, the PD-1 gene expression was highly expressed in high ARGscore group (Fig. 4F). The above results suggest that high ARGscore may account for the worse prognosis of patients in the ARGs.cluster.B group.

To further explore the relationship between ARGscore and tumor immunology, we performed TIP analysis. To further explore the relationship between ARGscore and tumor immunology, we performed TIP analysis. The results suggest that steps 1, 2, 3 and 4 were more abundant in high ARGscore group, whilesteps 6 and 7 were more abundant in low ARGscore group. This result indicated that although high ARGscore group was helpful for initiation and processing phases of immune response, effective antitumor immunity was still suppressed (Fig. 4G).

**Fig. 5:**
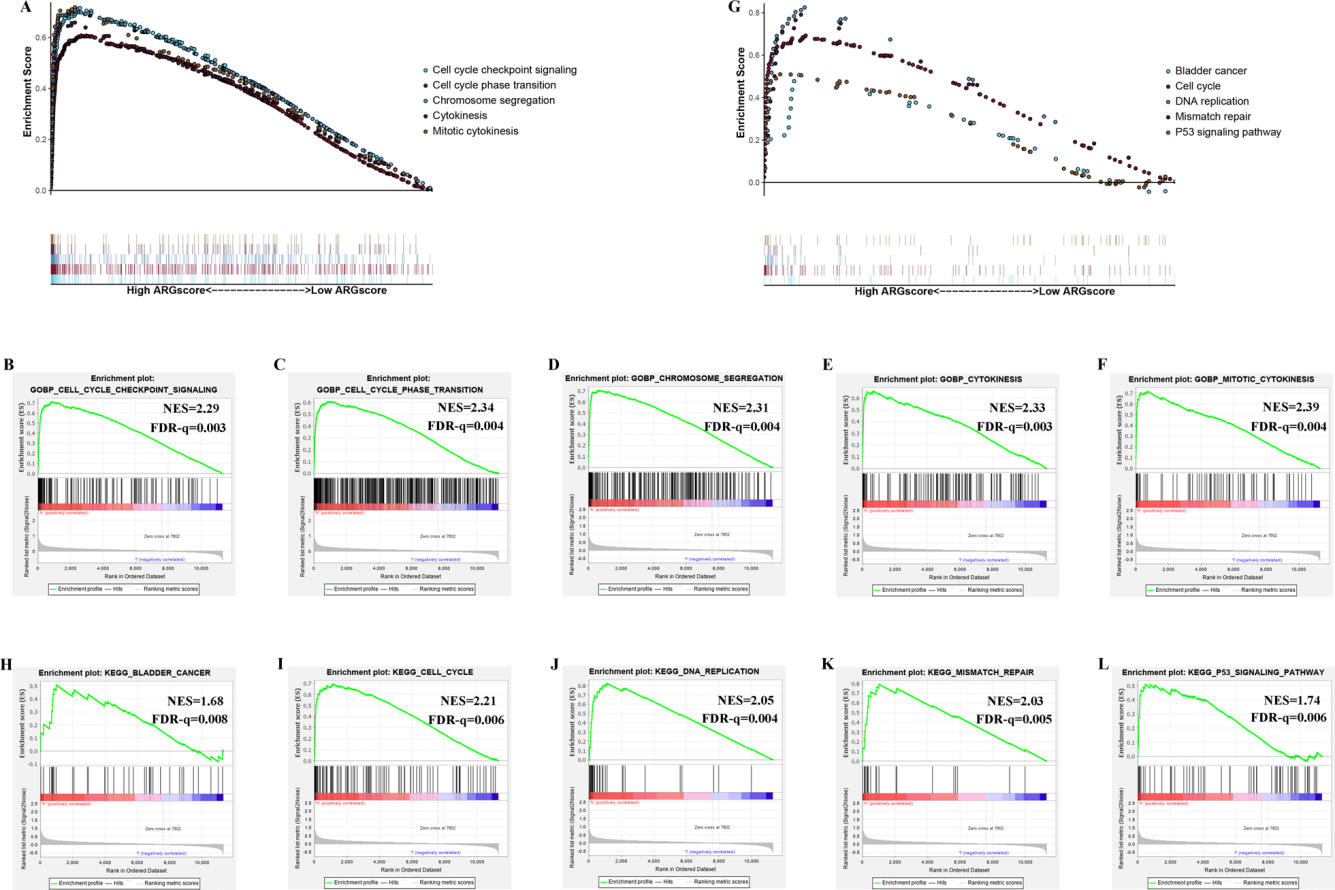
GSEA on the meta cohort to explore mechanisms underlying ARGscore. **A**–**F** GSEA GO identified high and low ARGscore groups related signaling pathways in BLCA. **G**–**L** GSEA KEGG identified high and low ARGscore related signaling pathways in BLCA

The results also showed that cell cycle-related pathways (cell cycle checkpoint signaling, cell cycle phase transition, chromosome segregation, cell cycle) and tumor-related pathways (bladder cancer, DNA replication, P53 signaling pathway) were enriched in the high ARGscore group (Fig. 5A–L). These results suggested that high ARGscore may have a significant impact in tumor development.

### ARGscore is a robust prognosis factor in BLCA

**Fig. 6:**
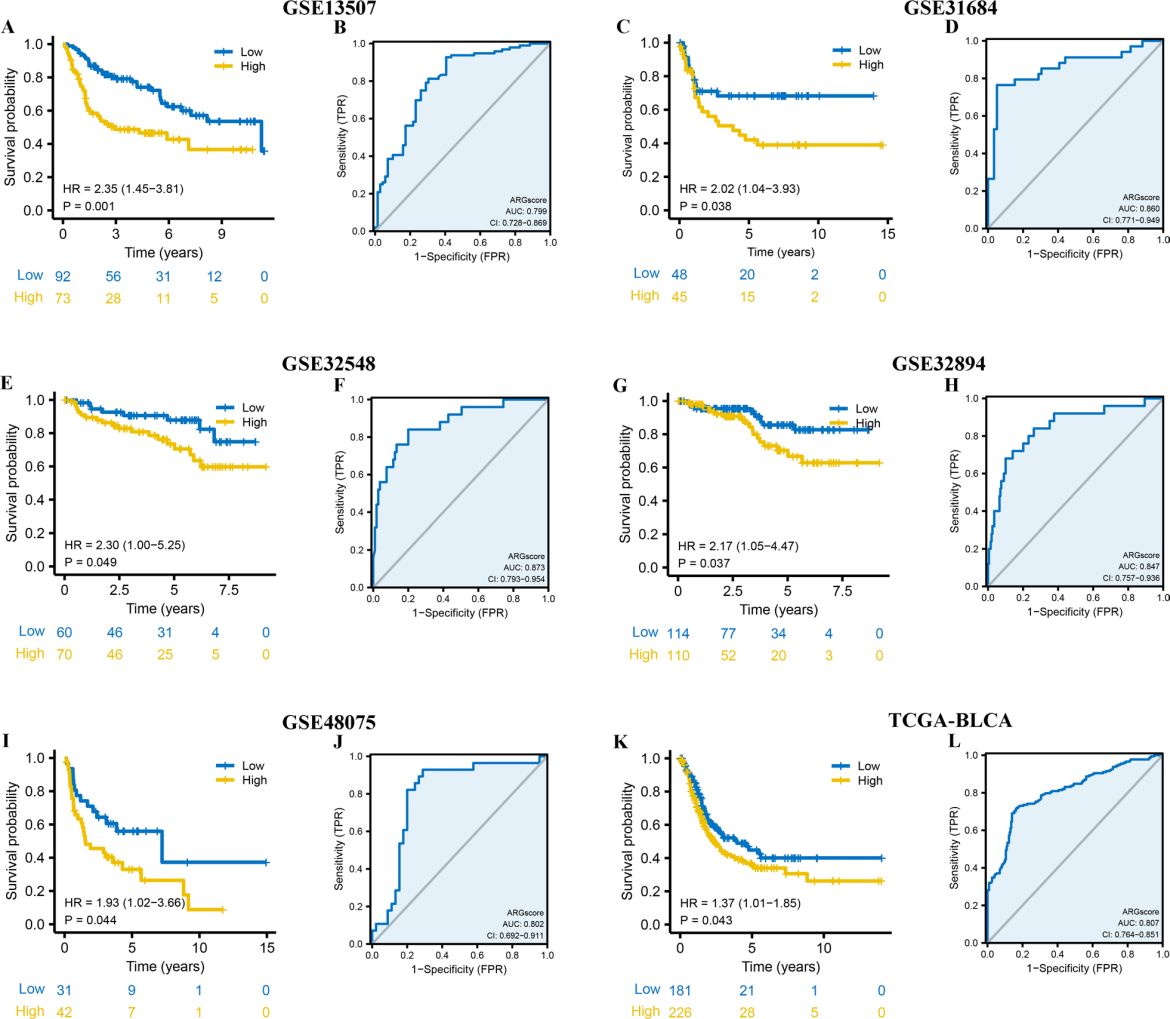
Internal and external validation of ARGscore. **A** Kaplan–Meier curves for high and low ARGscore groups in GSE13507. **B** The predictive value of ARGscore in GSE13507. **C** Kaplan–Meier curves for high and low ARGscore groups in GSE31684. **D** The predictive value of ARGscore in GSE31684. **E** Kaplan–Meier curves for high and low ARGscore groups in GSE32548. **F** The predictive value of ARGscore in GSE32548. **G** Kaplan–Meier curves for high and low ARGscore groups in GSE32894. **H** The predictive value of ARGscore in GSE32894. **I** Kaplan–Meier curves for high and low ARGscore patient groups in GSE48075. **J** The predictive value of ARGscore in GSE48075. **K** Kaplan–Meier curves for high and low ARGscore patient groups in TCGA-BLCA. **L** The predictive value of ARGscore in TCGA-BLCA

To further validate the robustness of ARGscore, the prognostic implication of ARGscore was examined in multiple independent datasets. In both patient sets, patients in the high ARGscore group had a worse OS than in the low ARGscore group (GSE13507: HR = 2.35, 95% CI = 1.45–3.81, P = 0.001, GSE31684: HR = 2.02, 95% CI = 1.04–3.93, P = 0.038, GSE32548: HR = 2.30, 95% CI = 1.00–5.25, P = 0.049, GSE32894: HR = 2.17, 95% CI = 1.05–4.47, P = 0.037, GSE48075: HR = 1.93, 95% CI = 1.02–3.66, P = 0.044, TCGA-BLCA: HR = 1.37, 95% CI = 1.01–1.85, P = 0.043) (Fig. 6A, C, E, G, I, K). In addition, the ROC curve analysis results demonstrated that the AUC of ARGscore in the GSE13507, GSE31684, GSE32548, GSE32894, GSE48075, TCGA-BLCA set were 0.799, 0.860, 0.873, 0.847, 0.802, and 0.807, respectively (Fig. 6B, D, F, H, J, L).

### High ARGscore suppress anti-PD1/L1 immunotherapy

**Fig. 7:**
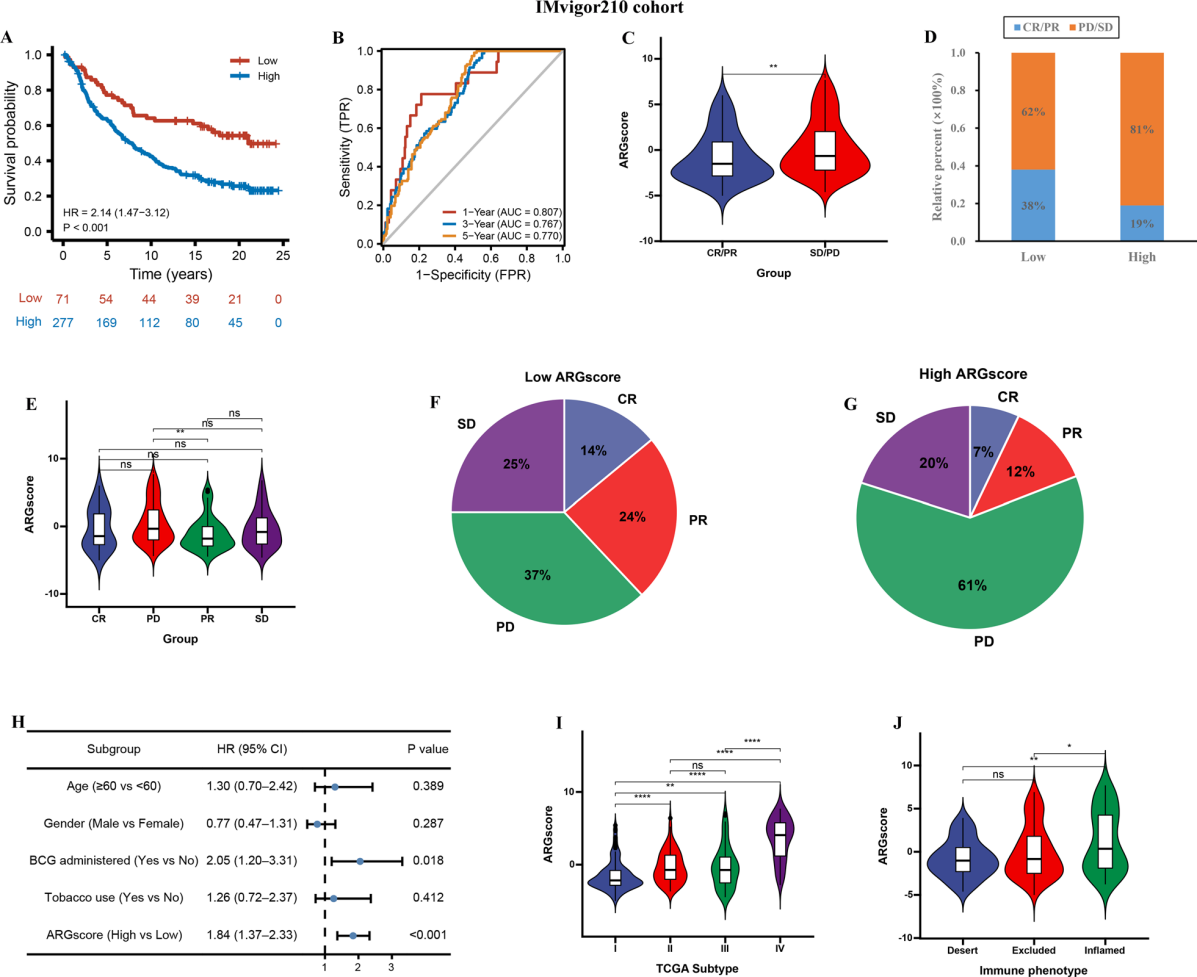
ARGscore in the role of anti-PD-1/L1 immunotherapy. **A** Survival analyses for low and high ARGscore groups in IMvigor210 cohort. **B** The predictive value of ARGscore in IMvigor210 cohort. **C** Differences in ARGscore among distinct anti-PD-1 clinical response groups. **D** The proportion of patients with response to PD-L1 blockade immunotherapy in low or high ARGscore groups. **E** Distribution of ARGscore in distinct anti-PD-L1 clinical response groups. **F**, **G** The number of CR, PR, PD, and SD patients in high and low ARGscore groups. **H** Multivariate Cox regression analysis for ARGscore in IMvigor210 cohort shown by the forest plot. **I**, **J** Differences in ARGscore between immune subtypes and TCGA subtypes

To further validate the robust of ARGscore in immunotherapy, 2 cluster in IMvigor210 cohort was identified by consensus clustering algorithm (Additional file 8: Fig. S4A–F). Then, a ARGscore was constructed based on 13 prognostic ARGs-related DEGs (Additional file 6: Table S6). Patients with high ARGscore had a worse OS (HR = 2.14, 95% CI = 1.47–3.12, P < 0.001, Fig. 7A). The AUC values 1, 3, and 5-survival rates in the cohort were 0.807, 0.767, 0.770, suggesting ARGscore had a good predictive performance (Fig. 7B). The ARGscore was lower in CR/PR group than in PD/SD groups (Fig. 7C). Moreover, the number of CR/PR patients was markedly higher in low ARGscore group than that in high ARGscore group (38% vs. 19%) (Fig. 7D, F, G), suggesting low ARGscore was sensitive to immunotherapy than high ARGscore group. The low ARGscore group identified twice as many patients as the high ARGscore group who responded to immunotherapy. In addition, ARGscore was lower in CR and PR group than in PD and SD groups (Fig. 7E). Multivariate Cox indicated also indicated that ARGscore could be acted as independent risk factor of BLCA patients in IMvigor210 cohort (HR = 1.84, CI = 1.37–2.33, P < 0.001) (Fig. 7H, Additional file 7: Table S7). The results showed that ARGscore was lower in desert group, TCGA I and II subtype than that other groups (Fig. 7I, J).

### Validation 4 ARGs in BLCA tissues

**Fig. 8:**
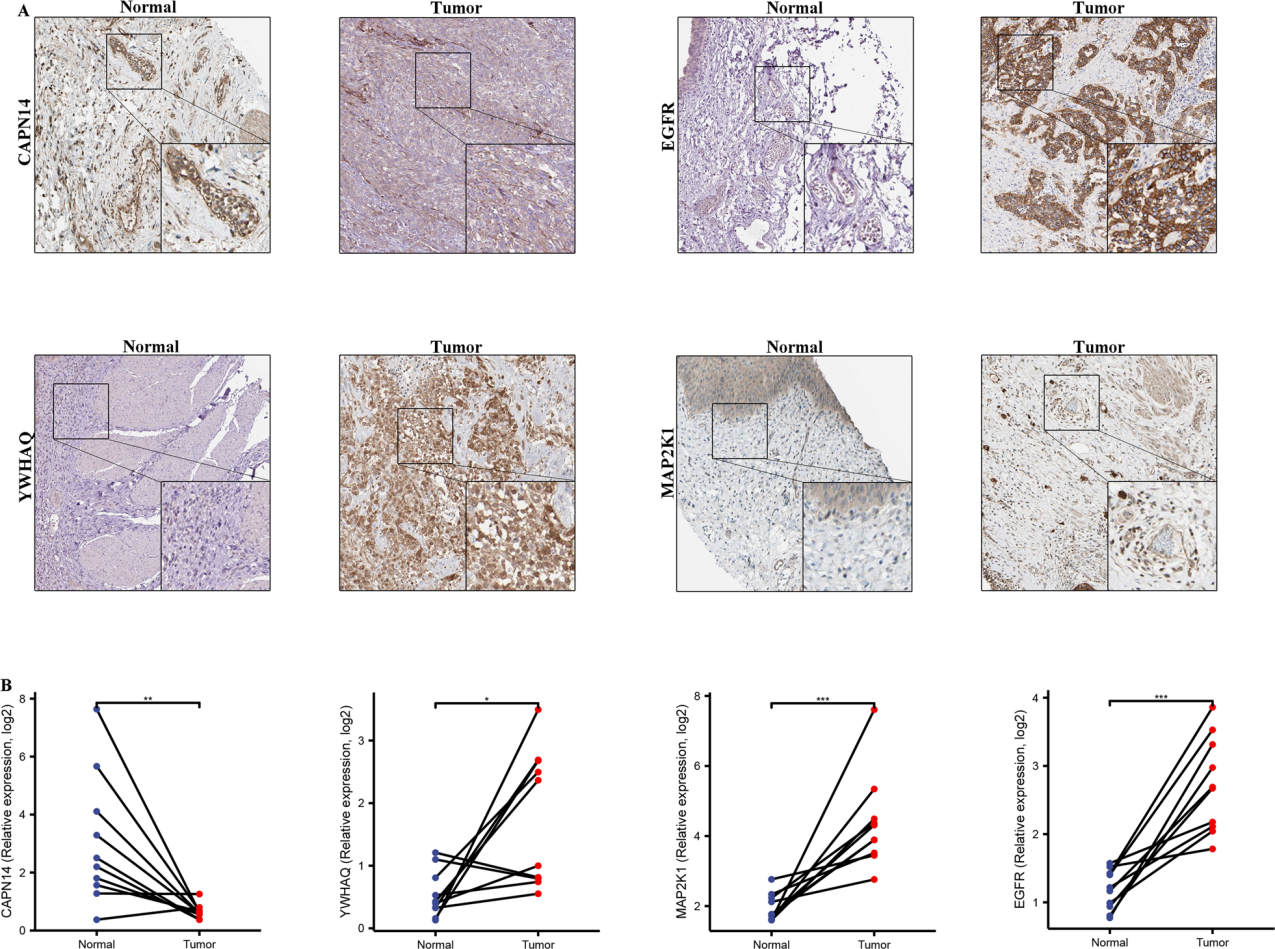
Representative immunohistochemistry images and qPCR of 4 ARGs expression in normal tissues and BLCA tissue. (**A**) Immunohistochemistry of 4 ARGs expression in normal tissues and BLCA tissue. (**B**) qPCR of 4 ARGs expression in normal tissues and BLCA tissue

We used IHC and qPCR to determine the protein level and mRNA expression of 4 ARGs with a tissue array of 10 BLCA patient samples. The IHC and qPCR result showed the expression of *EGFR, YWHAQ,* and *MAP2K1* were significantly higher in BLCA than in their corresponding adjacent nontumor tissues, *CAPN14* was significantly lower in BLCA than in their corresponding adjacent nontumor tissues (Fig. 8A, B).

## Discussion

Currently, chemotherapy remains one of the main treatment modalities for the vast majority of patients with advanced cancer, but tumor drug resistance has become a bottleneck limiting the efficacy of chemotherapy. Cell death can overcome the resistance of tumor cells to conventional chemotherapeutic drugs and promote the clearance of defective cells. Therefore, the induction of apoptosis in tumor cells may be a new approach for tumor treatment. Chen et al. revealed that guggulsterone inhibits BLCA growth and metastasis through caspase-dependent apoptosis [37]. Yin et al. showed that epigallocatechin-3-gallate can inhibited bladder cancer cells via inducing autophagy-related apoptosis [38]. We constructed a ARGscore prognostic model based on 1092 BLCA patients. Subsequently, the role of TME in BLCA was explored. This study revealed potential biomarkers and therapeutic targets in apoptosis-related signaling pathways.

In this study, we first downloaded transcriptomic information and clinical data of BLCA patients from the TCGA database. By comparing the expression of 91 ARGs in the tumor group and normal group, we showed that CAPN14, EGFR, YWHAQ, MAP2K1 were prognostic predictors of BLCA by univariate Cox regression analysis, and high expression of EGFR, YWHAQ, MAP2K1 were associated with poor prognosis of BLCA, low expression of CAPN14 was associated with poor prognosis of BLCA. Then, we divided BLCA into two molecular subtypes based on the expression of 4 ARGs. Cluster.B group had a worse prognosis compared to cluster.A, and patients in this group had a higher immune infiltration and higher ICI expression. This type is known as immunity tidal model type. Next, ARGscore model was constructed based on prognostic ARGs-related DEGs. ROC curve analysis confirmed that the model showed good predictive power for patients with BLCA.

Tregs and macrophage M2 have been shown to exert pro-tumor functions, while activated NK cells have the potential to kill tumor cells in different ways and are important mediators of cancer immune detection. In addition, Th1 and Th2 balance is associated with anti-tumor immunity, and in breast cancer patients, Th1/Th2 imbalance and elevated cytokine release from Th2 have been observed, and Caihu saponin can inhibit breast cancer growth by shifting Th1/Th2 balance to Th1 [39–41]. In the present study, immunosuppressive cells such as Tregs, Th2 and macrophages were more abundant in the high ARGscore group with poorer prognosis. The results suggest that the model is to some extent related to the immune landscape of the BLCA microenvironment, and the ARGscore model we constructed may play a key role in immune cell infiltration in BLCA.

M2-like macrophages, a major part of TAMs, have a strong influence on the formation of the immunosuppressive microenvironment [42, 43]. TAMs secrete a variety of growth factors, pro-inflammatory factors and enzymes. IL-6 and TNF-α can activate signaling pathways such as signal transduction molecules Smad and Wnt, which in turn activate transcription factors Snail family and Twist, prompting tumor cells to shift from epithelial to mesenchymal cell type, leading to a decrease in intercellular adhesion, shedding of tumor cells into the interrogative stroma and gaining the ability to easily metastasize to distant sites [44]. In vitro experiments showed that culturing BLCA cells with medium culturing M2-type macrophages resulted in activation of the *TLR4/STAT3* signaling pathway in BLCA cells, increased expression levels of EMT markers, and enhanced metastatic ability of tumor cells [45].

In recent years, ICIs have significantly transformed oncology treatment with the rise of immune checkpoint blockade therapies. Advances in checkpoint blockade therapies have deepened our understanding of the interactions between the immune system, cancer cells, and the tumor microenvironment. Tumor mutational burden and immune checkpoint-associated gene expression do not accurately predict response to ICIs therapy. Therefore, identifying biomarkers that can accurately predict the clinical efficacy of ICIs therapies is important to further advance precision immunotherapy. Among the many immunotherapeutic strategies, immune checkpoint inhibitors have shown great benefits in the treatment of a range of cancer types, such as the use of *CTLA-4* and *PD-1* or *PD-L1* to enhance anti-tumor immunity. At present, the biomarkers of ICIs therapy that show the most promise are *PD-L1* expression and tumor mutation burden (TMB), and microsatellite instability (MSI) [46, 47]. However, each of these biomarkers has certain limitations. The expression of PD-L1 is regulated by many mechanisms, and as a result, it has low reliability since there are often great differences in its expression at different times and conditions. Meanwhile, the positivity rate of *PD-L1* in BLCA was less than 10% [48]. Study also showed a significant correlation between positive expression of *PD-L1* and immune efficacy of immunotherapy. TMB is greatly influenced by the equipment of different laboratories, and the level of TMB in BLCA was not significant [49]. In contrast, MSI or MMR occurs in only 2–3% of BLCA patients [50]. Through our research, we have confirmed the influence of ARGscore on the prognosis and immunotherapy of BLCA patients who received ICIs treatment. Taking the expression of 4 ARGs as the significant criteria, this paper expounds the influence of ARGscore on the immune microenvironment and immunogenicity, so as to create accurate guidelines for the use of ICIs treatment in BLCA patients. The low ARGscore group identified twice as many patients as the high ARGscore group who responded to immunotherapy.

The present study also has some limitations; our study is based on publicly mined databases, no clinical experimental studies have been conducted, and their role and function in apoptosis processes are yet to be validated using more advanced methods and techniques. Second, this study was retrospective, so the risk score model needs to be further validated in prospective studies and multicenter clinical trials.

## Conclusion

In summary, we identified that *SCAPN14, EGFR, YWHAQ, MAP2K1* could be used as prognostic factors for BLCA by comprehensive analysis of ARGs. Meanwhile, 2 molecular subtypes were constructed based on 4 ARGs, and ARGscore was constructed based on the ARGs-related DEGs between subtypes. ARGscore can be used as BLCA independent predictor with good predictive ability. Also, high ARGscore group was sensitive to immunotherapy. In conclusion our study provides a promising avenue for individualized survival prediction and clinical outcome prediction of ICIs antitumor immunotherapy in patients with BLCA.

## Supplementary Information

**Additional file 1. Supplementary table 1.** Clinical characteristics of the BLCA patients used in this study.

**Additional file 2. Supplementary table 2.** List of 91 apoptosis related genes used in this study.

**Additional file 3. Supplementary table 3.** Differentially expressed apoptosis related genes between tumor and normal tissue inTCGA-BLCA dataset.

**Additional file 4. Supplementary table 4.** The results of the univariate Cox regression analysis between gene expression and OS inTCGA-BLCA dataset.

**Additional file 5. Supplementary table 5.** List of 2859 ARGs phenotype-related prognostic genes used in this study.

**Additional file 6. Supplementary table 6.** List of 13 ARGs phenotype-related prognostic genes used in this study in IMvigor210cohort.

**Additional file 7. Supplementary table 7.** Summary of detailed clinical information and apoptosisscore in IMvigor210 (mUC) cohort.

**Additional file 8. Supplementary figure 1.** (**A**-**D**) The relationship between four genes and immune infiltration. **Supplementary figure 2.** (**A**-**F**) Unsupervised consensus clustering based on 4 ARGs prognostic genes in a meta cohort. **Supplementary figure 3.** Differences in checkpoint expression between ARGs.cluster.A and ARGs.cluster.B groups. **Supplementary figure 4.** (**A**–**F**) Unsupervised consensus clustering based on 4 ARGs in IMvigor210 cohort.

## Data Availability

All data used in the study can be downloaded from the TCGA data repository (https://portal.gdc.cancer.gov/) and the GEO database (https://www.ncbi.nlm.nih.gov/geo/).
