# Supplementary material for: Apoptosis related genes mediated molecular subtypes depict the hallmarks of the tumor microenvironment and guide immunotherapy in bladder cancer
Source: BMC Med Genomics. 2023 Apr 28;16:88. doi: 10.1186/s12920-023-01525-8 (PMC10148450; doi:10.1186/s12920-023-01525-8)

Supplementary figure 1. (A-D) The relationship between four genes and immune infiltration


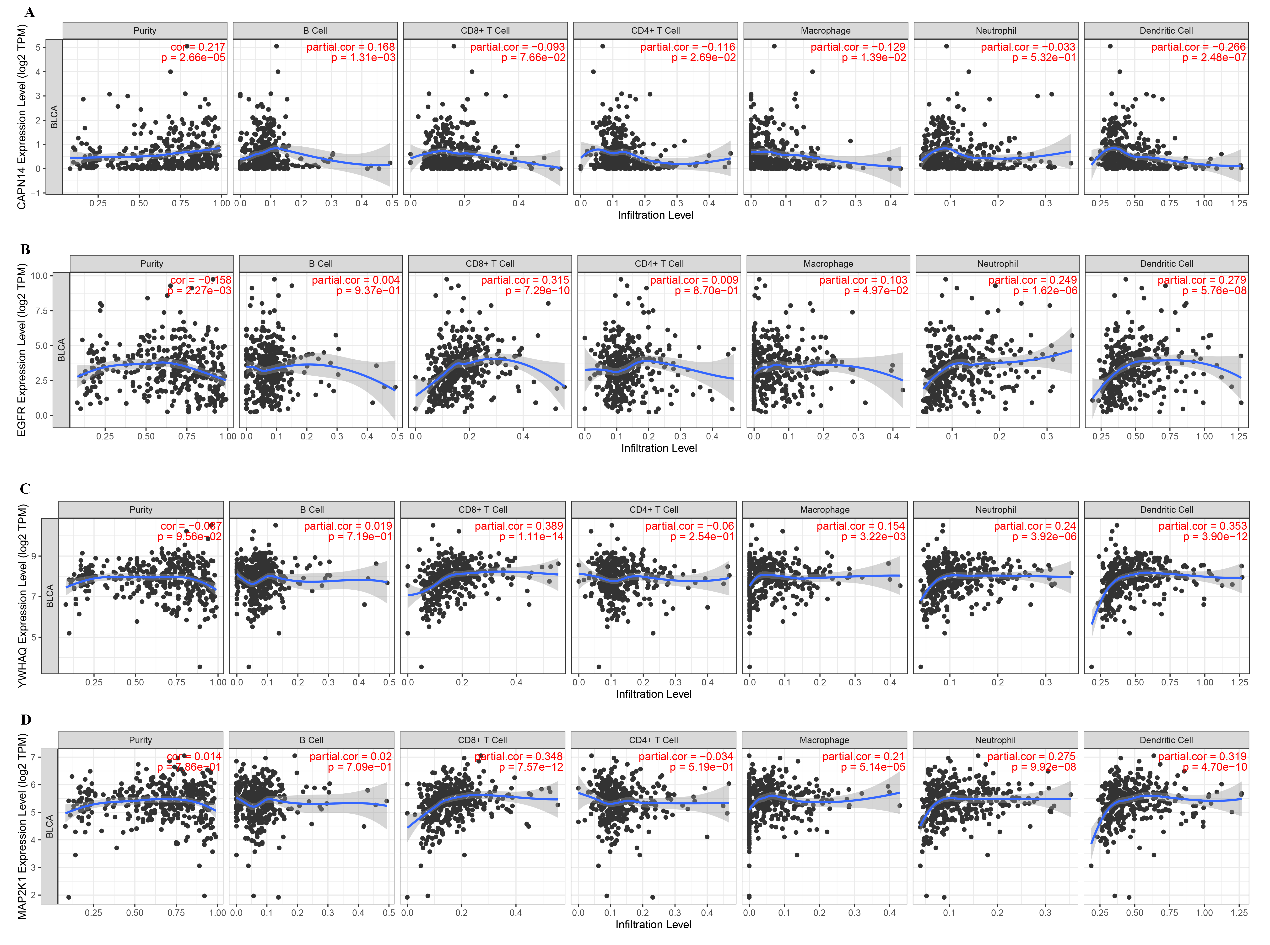


Supplementary figure 2. (A-F) Unsupervised consensus clustering based on 4 ARGs prognostic genes in a meta cohort.


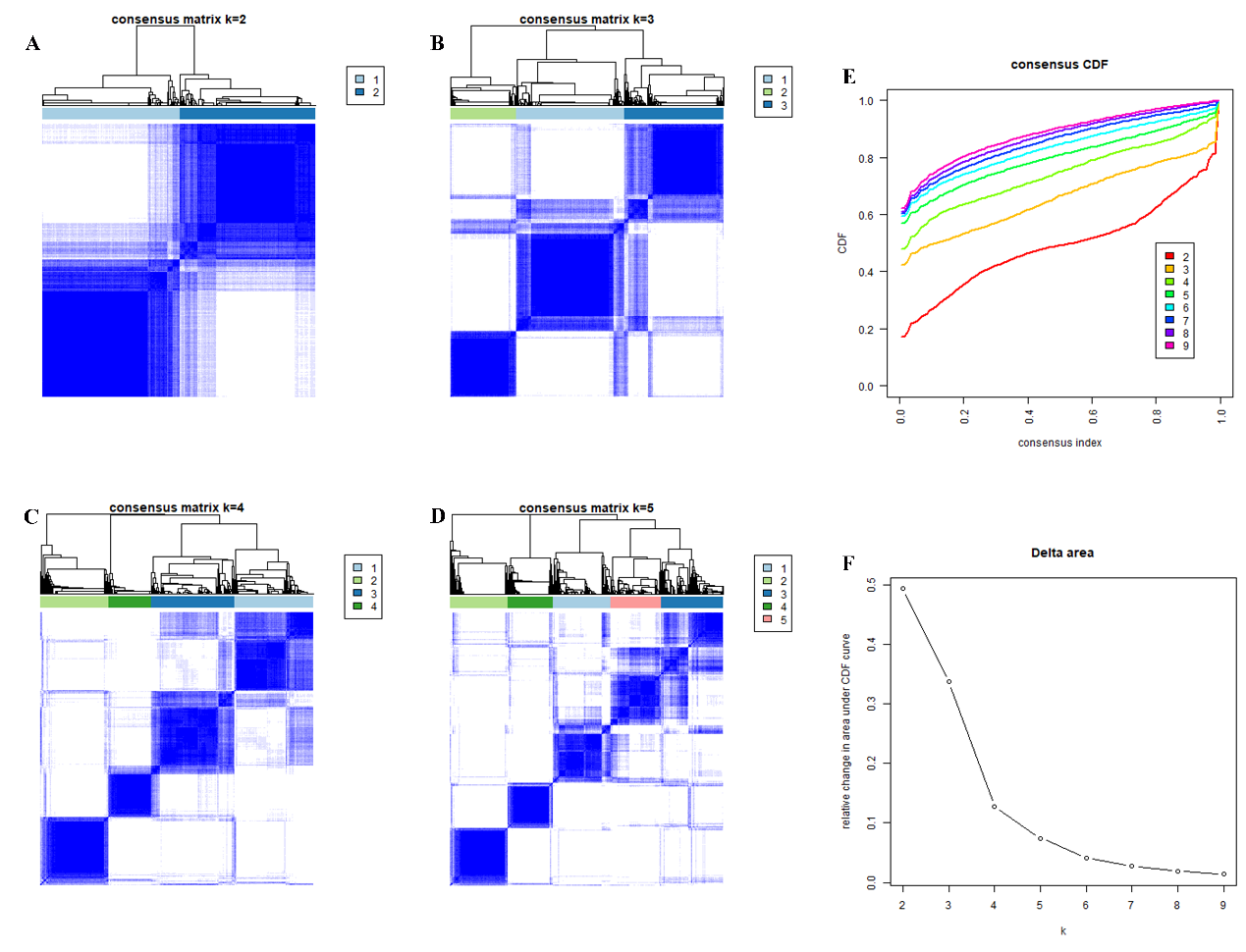


Supplementary figure 3. Differences in checkpoint expression between ARGs.cluster.A and ARGs.cluster.B groups.


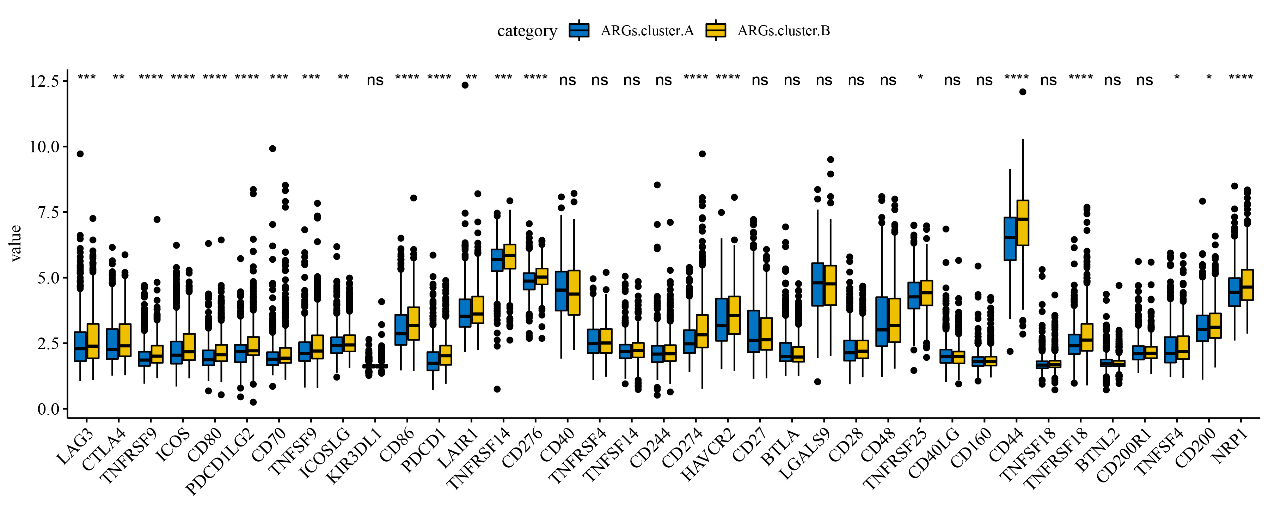


Supplementary figure 4. (A-F) Unsupervised consensus clustering based on 4 ARGs in IMvigor210 cohort.


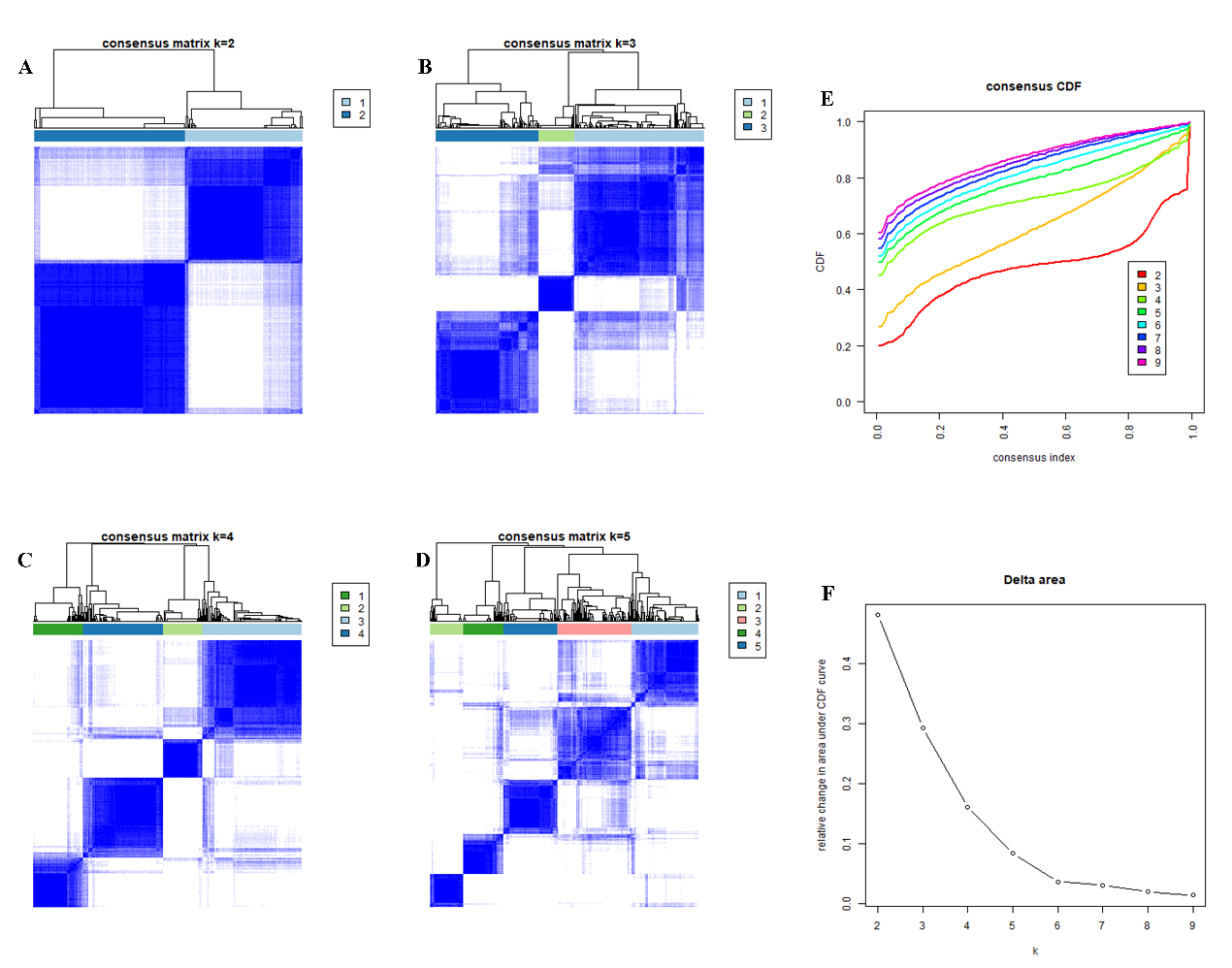

Supplement: Supplementary file 8 — Additional file 8. Supplementary figure 1. (A-D) The relationship between four genes and immune infiltration. Supplementary figure 2. (A-F) Unsupervised consensus clustering based on 4 ARGs prognostic genes in a meta cohort. Supplementary figure 3. Differences in checkpoint expression between ARGs.cluster.A and ARGs.cluster.B groups. Supplementary figure 4. (A–F) Unsupervised consensus clustering based on 4 ARGs in IMvigor210 cohort. [file 12920_2023_1525_MOESM8_ESM.docx]
